# Comment on Villalva et al. Antioxidant, Anti-Inflammatory, and Antibacterial Properties of an *Achillea millefolium* L. Extract and Its Fractions Obtained by Supercritical Anti-Solvent Fractionation against *Helicobacter pylori*. *Antioxidants* 2022, *11*, 1849

**DOI:** 10.3390/antiox12061226

**Published:** 2023-06-07

**Authors:** Rafał Frański, Monika Beszterda-Buszczak

**Affiliations:** 1 Faculty of Chemistry, Adam Mickiewicz University, Uniwersytetu Poznańskiego 8, 61-614 Poznań, Poland; 2 Department of Food Biochemistry and Analysis, Poznań University of Life Sciences, Mazowiecka 48, 60-623 Poznań, Poland

**Keywords:** yarrow, flavonoids, fragmentation pathway, mass spectrometry, electrospray ionization

## Abstract

Villalva et al. evaluated the potential utility of an *Achillea millefolium* (yarrow) extract in the control of *H. pylori* infection. The agar-well diffusions bioassay was applied to determine the antimicrobial activity of yarrow extracts. The supercritical anti-solvent fractionation process of yarrow extract was made to give two different fractions with polar phenolic compounds and monoterpenes and sesquiterpenes, respectively. Phenolic compounds were identified by HPLC-ESIMS by using the accurate masses of [M−H]^−^ ions and the characteristic product ions. However, some of the reported product ions seem to be disputable, as described below.

Eradication of *Helicobacter pylori* has become a serious challenge due to increasing antimicrobial resistance. That Gram-negative, microaerophilic bacterium that is known to affect over 50% of the worldwide population occurs initially during childhood but, if left untreated, could persist for life, resulting in a broad spectrum of gastropathies. It is particularly important because *H. pylori* is involved in the development of 80% of gastric cancers and 5.5% of all malignant conditions worldwide [1,2,3]. 

The most effective conventional therapies for *H. pylori* infection require a minimum of two antibiotics (commonly, amoxicillin and clarithromycin) in combination with a gastric acid inhibitor or bismuth to guarantee high eradication rates. That is why nowadays, much research work is concentrated on the search for new potential anti-*H. pylori* candidates, among others, vegetables and plant extracts showing antibacterial properties [4,5]. They act by inhibiting bacterial enzymes, suppressing nuclear factor-κB, adhesions with gastric mucosa, and by inhibiting oxidative stress [6,7]. Organic extracts (ethanol, methanol, acetone, chloroform, petroleum ether, and mixtures of mentioned) of *Acacia nilotica*, *Alchornea triplinervia*, *Arrabidaea chica*, *Bridelia micrantha*, *Calotropis procera*, *Camellia sinensis*, *Carum carvi*, *Cocculus hirsutus*, *Derris trifoliate*, *Geranium wilfordii*, *Hydrastis canadensis*, *Myristica fragrans*, *Xanthium brasilicum*, and *Trachyspermum copticum* and many others have demonstrated antibacterial activity against clinical isolates of *H. pylori* [8,9,10,11,12,13,14,15]. In the study by Villalva and coworkers, the potential utility of an *Achillea millefolium* (yarrow) extract in the control of *H. pylori* infection was evaluated [16]. The supercritical anti-solvent fractionation process of yarrow extract was made to give two different fractions with polar phenolic compounds and monoterpenes and sesquiterpenes.

Yarrow (*Achillea millefolium* L.) is one of the most commonly used medicinal herbs, in both folk and conventional medicine, for over 3000 years, growing wild and as a cultivated plant in the region of Eurasia and North America [17]. According to the conducted studies, *Achillea millefolium* L. is a biologically active plant that demonstrates multiple beneficial effects, including antioxidant, anti-inflammatory, spasmolytic, diaphoretic, hepatoprotective, choleretic, antipyretic, analgesic, antimicrobial and anticancer properties [18,19,20]. *Achillea millefolium* L. helps eliminate toxins from the body, controls bleeding, lowers blood pressure, relieves menstrual pain, and is used in the treatment of various diseases such as diabetes, tuberculosis, Alzheimer’s, and Parkinson’s disease [21,22,23]. Moreover, as indicated by Tilwani and coworkers, the yarrow-treated SARS-nCoV-2 cell exhibits the disintegration of the virus membrane [24].

Different extracts (hexane, petroleum ether, and methanol) of *A. millefolium* aerial parts were found to be active toward the following pathogens: *Bacillus cereus*, *Staphylococcus aureus*, *Escherichia coli*, *Klebsiella pneumoniae*, *Pseudomonas aeruginosa*, *Salmonella enteritidis*, *Yersinia enterocolitica*, *Aspergillus niger*, and *Candida albicans* [17,25,26]. As the literature indicates, the Gram-positive bacteria were more susceptible than the Gram-negative ones, whereas *S. aureus* and *B. cereus* were the most susceptible Gram-positive bacteria [25,27].

In the study by Villalva et al., the agar-well diffusions bioassay was applied to determine the antimicrobial activity of yarrow extracts (YE). YE was significantly (*p* < 0.05) effective as an antibacterial agent against all *H. pylori* strains tested (Hp48, Hp53, and Hp59) in the range of CFU reduction between 4.8 and 7.1 log [16]. Moreover, even better results were obtained for the fraction enriched in volatile compounds. In turn, in the experiment with the inflammatory response induced by *H. pylori* in AGS cells, interleukin 8 (IL-8) has been used as a biomarker. It is a well-documented fact that *H. pylori* infection is associated with an increase in IL-8 concentration, both in vitro and in vivo; it was among the first cytokines described to be produced by infected gastric epithelium; and leads to the recruitment of leukocytes in the gastric mucosa, representing a major step in the regulation of immune-inflammatory responses [2]. In the study by Villalva et al., the application of YE decreased IL-8 synthesis by 53% to 64% in human gastric epithelial cells, with a suggestion that the two types of compounds could contribute to this inhibition—not only phenolic compounds but also some essential oils. In addition, the authors analyzed the antioxidant activity of YE and its fractions against intracellular reactive oxygen species (ROS) synthesis in *H. pylori*-infected AGS cells. The inhibition effect of YE on ROS production depended on the examined *H. pylori* strain and ranged from 16% to 29%, while the fraction enriched in phenolic compounds was more active, regardless of the strain used, with about 40% intracellular ROS inhibition.

In the study by Villalva et al., the volatile compounds have been identified by GC-MS, and only retention times have been provided for them (Table 2 of [16]). Phenolic compounds have been identified by HPLC-ESIMS in the negative ion mode, and aside from the retention times, the accurate masses of [M−H]^−^ ions and the characteristic product ions (MS/MS ions) have also been provided (Supplementary Material, Table S1 of [16]). However, some of the product ions seem to be disputable, as described below.

The most disputable is the product ion at *m*/*z* 112 reported for apigenin and diosmetin. This is the only product ion reported for these two compounds and has 100% ri (Supplementary Material, Table S1 of [16]). According to the published data, apigenin should yield characteristic product ions at *m*/*z* 225, 151, 149, 117, and 107, although the relative abundances of these ions may be different, depending on the instrumental conditions used [28,29,30,31]. Diosmetin should yield an abundant product ion at *m*/*z* 284 as a result of methyl radical loss—a characteristic feature of methoxylated flavonoids [32,33]. The other characteristic diosmetin product ions should be at *m*/*z* 256, 227, 151, and 107 [34]. Besides diosmetin, Villalva et al. have found one other methoxylated flavonoid, named ‘methoxyquercetin isomer’ (the third most abundant flavonoid in yarrow’s precipitated fraction, Table 1 of [16]); however, taking into account the *m*/*z* of [M−H]^−^ at 315, it should be *O*-methyl quercetin. Villalva et al. claim only the detection of a product ion at *m*/*z* 301, thus the loss of mass 14 (elimination of a CH_2_ moiety). As mentioned above, the characteristic feature of the fragmentation of [M−H]^−^ ions of methoxylated flavonoids is the loss of mass 15 (loss of a methyl radical). Furthermore, other product ions should be observed as well, enabling at least tentative identification of this compound (isorhamnetin glycosides have already been found in the *Achillea millefolium* L. [35,36]). It has to be stressed that the two other methoxylated flavonoids, centaureidin and casticin, have been observed to have methyl radical losses by Villalva et al. [16]. The authors claim the detection of one biflavonoid, namely amentoflavone ([M−H]^−^ at *m*/*z* 537), for which they have reported two product ions at *m*/*z* 519 and 495. However, the characteristic amentoflavone product ions are at *m*/*z* 443, 417, 375 (the most abundant), and 331 [37,38].

Villalva et al. have also detected a number of flavone C-glycosides. Among them are three isomers, namely apigenin-C-hexoside-C-pentoside, schaftoside, and schaftoside isomer ([M−H]^−^ at *m*/*z* 563). For all these three compounds, Villalva et al. have obtained two identical product ions with identical relative abundances (*m*/*z* 473 and 443, 10 and 20% ri, respectively). The loss of mass 90 and 120 is a characteristic feature of flavone C-glycosides fragmentation [39]; thus, the product ions at *m*/*z* 473 and 443 are typical of these compounds. However, other product ions should also be detected, e.g., at *m*/*z* 383 and 353, and at least minor differences in relative ion abundances should be observed for these three isomers detected [40,41]. Villalva et al. claim the detection of luteolin-6,8-di-C-glucoside ([M−H]^−^ at *m*/*z* 609), for which they have detected two product ions at *m*/*z* 489 and 325 [16]. The first one (loss of mass 120) is a characteristic product ion of luteolin-6,8-di-C-glucoside, often having 100% ri; however, at *m*/*z* 325 it is not the characteristic one. Other characteristic product ions which should be observed for this compound are at *m*/*z* 591, 399, 369, and 327 [41,42]. Villalva et al. claim the detection of vicenin 2 (apigenin 6,8-di-C-glucoside); however, no product ions have been reported for this compound [16]. For the two last flavone C-glycosides, namely homoorientin (luteolin 6-C-glucoside) and vitexin (apigenin 8-C-glucoside), the reported by Villalva et al. product ions match perfectly with those, the most abundant ones, reported elsewhere (although vitexin cannot be classified as flavonols) [43,44].

The product ions detected by Villalva et al. for other flavonoids are in agreement with those reported in the literature, at least as concerns the most characteristic ones. For example, for quercetin, the authors have detected only the product ion at *m*/*z* 151 [16]. Although deprotonated quercetin molecule yields a few other product ions (Figure 1), that at *m*/*z* 151 (formally ^1,3^A^−^ product ion) is the most abundant, formed through the retro-Diels–Alder reaction, and can be regarded as a diagnostic ion for 5,7-dihydroxyflavonoids [28,45,46].

Villalva et al. have also detected a number of hydroxycinnamic acids and their conjugates, and the detection of most of them does not raise any doubts. The only disputable ones are the product ions reported for chlorogenic acid (*trans*-5-*O*-caffeoylquinic acid) and its isomer, cryptochlorogenic acid (*trans*-4-*O*-caffeoylquinic acid). For chlorogenic acid and cryptochlorogenic acid, Villalva et al. have obtained two identical product ions of almost identical relative abundances, namely at *m*/*z* 191 (100% ri) and 161 (10–11% ri) [16]. However, the fragmentation patterns of these compounds should be significantly different. Chlorogenic acid yields abundant product ions at *m*/*z* 191, and other product ions have very low abundances (practically are not detectable), whereas cryptochlorogenic acid, except the product ion at *m*/*z* 191, yields abundant product ions at *m*/*z* 179, 173, and 135 [47,48,49,50]. On the other hand, it has to be stressed that the product ions detected by Villalva et al. for neochlorogenic acid (*trans*-3-*O*-caffeoylquinic acid) perfectly match those reported elsewhere with respect to their *m*/*z* and ri values [47,48,49]. The detection of other hydroxycinnamic acids and their conjugates also does not raise any doubts, although the accurate *m*/*z* of ferulic acid [M−H]^−^ ion should be 193.0504, not 103.0504 (minor typos error). It is also worth adding that the elution order of the mono-*O*-caffeoylquinic acid isomers ([M−H]^−^ at *m*/*z* 353) and di-*O*-caffeoylquinic acid isomers ([M−H]^−^ at *m*/*z* 515) reported by Villalva et al. perfectly matches that obtained elsewhere during reversed-phase liquid chromatographic analysis [51].

It should be emphasized that our very specific comments concerning the product ions do not affect the paper by Villalva et al., the paper is characterized by a high scientific level, and the authors finding may imply the next practical application of *Achillea millefolium* L. extracts. On the other hand, the correction of Table S1 is desirable, maybe as a reply to our comment or as a corrigendum to their paper since it surely would improve the quality of the paper.

## Figures and Tables

**Figure 1:**
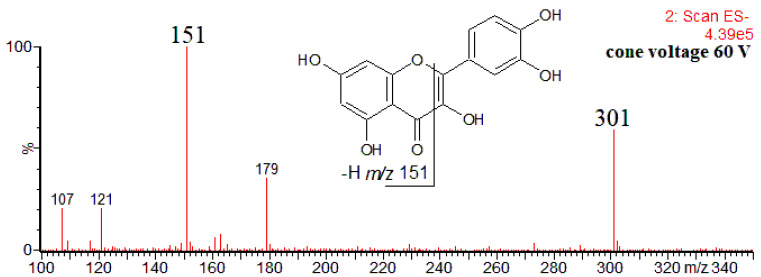
ESI mass spectrum (CID in-source) of quercetin obtained upon HPLC-MS(−) analysis of *Prunus persica* bark extract [46].
